# Natural Killer and T Cell Infiltration in Canine Osteosarcoma: Clinical Implications and Translational Relevance

**DOI:** 10.3389/fvets.2021.771737

**Published:** 2021-11-16

**Authors:** Aryana M. Razmara, Sean J. Judge, Alicia A. Gingrich, Sylvia M. Cruz, William T. N. Culp, Michael S. Kent, Robert B. Rebhun, Robert J. Canter

**Affiliations:** ^1^Department of Surgery, School of Medicine, University of California, Davis, Davis, CA, United States; ^2^Department of Surgical and Radiological Sciences, School of Veterinary Medicine, University of California, Davis, Davis, CA, United States

**Keywords:** osteosarcoma, NK cells, T cells, tumor microenvironment (TME), immunotherapy, canine model

## Abstract

Metastatic osteosarcoma has a bleak prognosis in both humans and dogs, and there have been minimal therapeutic advances in recent decades to improve outcomes. Naturally occurring osteosarcoma in dogs is shown to be a highly suitable model for human osteosarcoma, and limited data suggest the similarities between species extend into immune responses to cancer. Studies show that immune infiltrates in canine osteosarcoma resemble those of human osteosarcoma, and the analysis of tumor immune constituents as predictors of therapeutic response is a promising direction for future research. Additionally, clinical studies in dogs have piloted the use of NK transfer to treat osteosarcoma and can serve as valuable precursors to clinical trials in humans. Cytotoxic lymphocytes in dogs and humans with osteosarcoma have increased activation and exhaustion markers within tumors compared with blood. Accordingly, NK and T cells have complex interactions among cancer cells and other immune cells, which can lead to changes in pathways that work both for and against the tumor. Studies focused on NK and T cell interactions within the tumor microenvironment can open the door to targeted therapies, such as checkpoint inhibitors. Specifically, PD-1/PD-L1 checkpoint expression is conserved across tumors in both species, but further characterization of PD-L1 in canine osteosarcoma is needed to assess its prognostic significance compared with humans. Ultimately, a comparative understanding of T and NK cells in the osteosarcoma tumor microenvironment in both dogs and humans can be a platform for translational studies that improve outcomes in both dogs and humans with this frequently aggressive disease.

## Introduction

Osteosarcoma (OSA) is an aggressive cancer of the skeleton in both dogs and humans with high rates of metastasis. Untreated, 90% of dogs with OSA develop metastasis within 1 year, and 85–90% of humans do so within 2 years (1). When gross metastatic disease develops, survival is dismal, and fewer than 20% of human patients survive 5 years and fewer than 5% of dogs survive 2 years with disseminated disease (2, 3). In the past few decades, there has been limited advancement of OSA therapies, and outcomes for patients with metastatic disease have remained stagnant (4, 5). Canine OSA (cOSA) occurs spontaneously and shares notable genomic profiles, clinical presentations, and progression patterns with human OSA (hOSA) (1, 6–8). The intact immune system of dogs with naturally occurring cancer along with the relatively high incidence of cOSA and extensive similarities between cOSA and hOSA make companion dogs an ideal platform for translational oncology, especially in the investigation of novel immunotherapies (9, 10).

NK cells are innate immune cells with cytokine-producing and cytotoxic effector capabilities that have been identified in the OSA tumor microenvironment (TME) along with cytotoxic and helper T cells (11, 12). Both NK and CD8+ T cells have the capability to kill cancer cells using their cytotoxic functions, but their potential cooperation is complex. The downregulation of MHC-I by certain cancer cells effectively circumvents recognition by CD8+ T cells but simultaneously increases activation of NK cells by removing a major inhibitory signal (13). Additionally, IFN-γ secreted by NK cells stimulates CD4+ T cell activation and is required for proliferation of CD8+ T cell precursors (13). In many cancers, such as melanoma, gastric cancer, and myeloma, among others, secretion of IFN-γ is also shown to induce PD-L1 expression in tumor cells (14). IFN-γ-induced upregulation of PD-L1 expression on immune and tumor target cells is recognized as a conserved mechanism of adaptive immune resistance and tolerance as a response to chronic antigen stimulation, which is observed in both cancers and chronic pathogen exposure (15–17). These cooperative antitumor properties of NK and both CD4+ and CD8+ T cells are contrasted by studies showing that NK cells kill activated T cells to protect against virus-induced immunopathology (18, 19). Even among tumor-infiltrating T cells, tumor and immune cells expressing PD-L1 can inhibit neighboring PD-1+ T cells through the PD-1/PD-L1 axis, an immune checkpoint that cancer cells can exploit to inhibit antitumor immune responses (20). In humans, NK and T cells also show increased exhaustion markers in the solid TME, making reversal of the resulting immunosuppression a key aim of emerging immunotherapies (21). Veterinary studies also identify features of immune exhaustion in dogs with cancer (22, 23), but focused studies are needed to answer lingering questions of the consistency of these markers and how to target them. Analyses establishing the extent to which cOSA infiltrating NK and T cells are comparable to hOSA support a deeper understanding of the OSA TME and advance bench-to-bedside studies to speed the translation of novel immunotherapies. This review focuses on the recent literature characterizing NK and T cell infiltrates in OSA tumors and their prognostic significance in humans and dogs.

## Blood vs. Tumor

The TME is made up of tumor cells, healthy stromal and nonimmune cells, and immune cells, all of which are communicating in dynamic interactions that work both for and against the tumor (24). These interactions occur in the context of a systemic immune response, including immune cell activity within the peripheral circulation, which, interestingly, does not inherently parallel activity in the TME (25–29).

In healthy dogs, CD4+ and CD8+ T cells comprise approximately 49 and 22% of lymphocytes, respectively, in peripheral blood, and T regulatory cells (Tregs) account for 4.5% of CD4+ T cells (25). Walter et al. (12) looked at peripheral immune responses in dogs prior to and following chemotherapy and found that dogs with osteosarcoma have fewer pretreatment CD4+ and CD8+ T cells in the blood than healthy dogs. Canine Tregs have also been identified and found to be higher in blood from dogs with OSA compared with healthy dogs (25, 30, 31). Later, the same working group established the clinical relevance of circulating lymphocytes in cOSA. For example, Sottnik et al. (32) observed that dogs with lower monocyte counts and lymphopenia prior to treatment with amputation and adjuvant chemotherapy had an increased disease-free interval (DFI). The authors call attention to the fact that this contrasts with human studies in which lymphopenia is associated with worse outcomes in sarcomas and other cancers (33). However, recent hOSA studies largely focus on lymphocytes in the context of other blood parameters, such as high neutrophil-to-lymphocyte ratios (NLRs) or low lymphocyte-to-monocyte ratios (LMRs), which are both associated with poor prognosis (34, 35). The necessity of lymphocyte ratios could be explained by the importance of other immune cell populations and the conflicting functions of different lymphocyte subsets, such as Tregs. For example, Biller et al. (25) analyzed CD4+ T, CD8+ T, and Treg (defined as CD4+FOXP3+) cells by flow cytometry in cOSA and found that low circulating CD8/Treg ratios were associated with shorter survival time. Investigation of NLR and LMR within cOSA are needed for an accurate comparison of the prognostic significance of circulating lymphocytes in dogs.

Although circulating CD8/Treg ratios were associated with a significantly worse prognosis, this was not seen in cOSA tumor-infiltrating lymphocytes (TIL), an indication of the differing immune populations between blood and tumors (25). This discordance is further substantiated with evidence from the same study that Tregs are highest in cOSA tumors, making up 21% of lymphocytes in the TME, compared with Tregs in the lymph nodes and circulation (25). The pattern stays consistent in mouse and human OSA, where, compared with blood, tumors have a higher concentration of Tregs as well as more activated Tregs based on cellular proliferation and increased expression of activation markers (26). The similarities extend to other immune cell subsets. A recent comparative study by Judge et al. (27) observed that proportions of T and NK cells (using CD3, CD8, and NKG2D by PCR as readouts) were significantly higher in peripheral blood compared with the TME in both cOSA and human sarcomas. The authors also found that, though tumors have low infiltration of lymphocytes, activation and exhaustion markers of infiltrating CD8+ T and NK cells are higher than those found in circulation (28). In another study, CD3+ T cells in hOSA similarly had significantly higher expression of exhaustion markers than those in peripheral blood (29).

**Figure 1:**
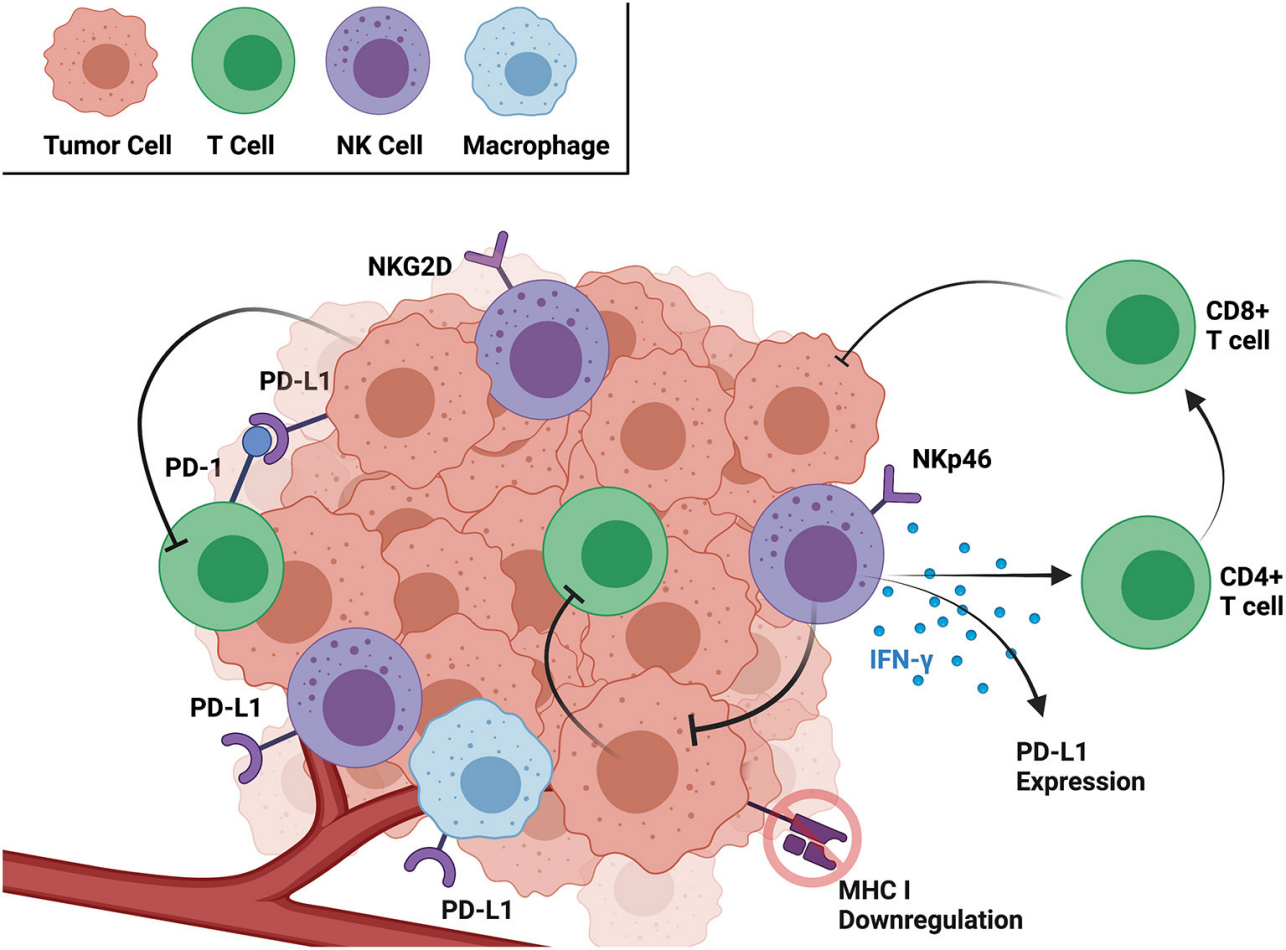
Interactions between cancer and immune cells within the TME as well as relevant receptors and soluble factors. Created with BioRender.com.

Based on the current literature, both human and dog OSA tumors contain CD3+ T, CD8+ T, and NK cells, and the activation and exhaustion of these immune cell subsets varies significantly between the tumor and circulation. The immune landscape of both the TME and peripheral circulation is important in identifying novel immunotherapies and patients most likely to respond to them (36). However, immunotherapies targeting immune cells in the TME, such as PD-1/PD-L1 inhibitors, have the added benefit of eliciting targeted antitumor responses, sometimes with minimal side effects (37). As a critical window into the mechanism of immune cell and solid tumor interaction, summarized in Figure 1, the remainder of this review focuses on the OSA TME specifically and characteristics of infiltrating T and NK cells.

## T Cells

Recent evaluation of cOSA tumors from our group using immunohistochemistry (IHC) confirmed minimal CD3 infiltration compared with normal lymph nodes (27). There was varied cOSA intra-tumoral CD3 and CD8 gene expression after radiotherapy (RT) plus NK transfer, which did not correlate significantly with survival, acknowledging that sample size was a limiting factor (27). However, these results suggest that an immune “cold” cOSA tumor could be transformed into a “hot” tumor with immunotherapy (27). This hypothesis stems from increasing studies of lymphocyte infiltration, or immune score, in human cancers with higher levels indicating hot tumors and those with low infiltration being cold tumors, which may be more accurate in predicting survival than the tumor-node-metastasis staging system (38). The ability to increase immune scores therapeutically is demonstrated by Modiano et al. (39), who found that the percentage of CD3+ T cells in cOSA jumps from 8 to 17% after fas-ligand gene therapy. The increase in TILs also correlates with survival because dogs with greater lymphocyte infiltration after treatment had longer survival times than dogs with lower infiltration (39). Similarly, in hOSA, CD8+ cells were observed in the majority of tumors but only made up 1% of intra-tumor cells (40). Even with low CD8+ staining within hOSA tumors, CD8+ cells were still significantly associated with improved prognosis and also favorably predicted survival posttreatment with zoledronic acid (40). These results together provide evidence of OSA being an immunologically cold tumor that can be treated to increase immune cell activity and improve survival.

On the other hand, some studies show cOSA to have varying patterns of TILs. Biller et al. (25) were among the first to evaluate tumor infiltrates of cOSA, finding that tumors were relatively highly infiltrated, made up of 19.2% CD4+ and 8.6% CD8+ T cells, but TILs were not associated with survival. The discrepancy may be due to varying techniques as this study determined percentage of cells by flow cytometric analysis of strained tumor samples rather than IHC evaluation. But Withers et al. (41) later also showed evidence of varying degrees of infiltration using IHC with CD3+ cells ranging from 4.6 to 607.6 cells/mm^2^ in cOSA tumors. Although CD3+ infiltrates alone were not prognostic, increased infiltration of CD204+ macrophages was associated with increased DFI, leading the authors to suggest that cOSA is an immunogenic tumor (41). In a second study, Withers et al. (42) further examined heterogeneity of infiltrates by comparing infiltrates within matched primary and metastatic cOSA tumors. They reported that overall immune infiltrates of the primary tumor correlated with a patient's metastatic lesions, but importantly, they also found that CD3+ and CD204+ macrophages were significantly higher in metastatic lung lesions compared with their primary tumor (42). The range of TILs in cOSA and inconsistent associations with survival, rather than conflicting each other, may point to intra-tumoral heterogeneity within cOSA and complicate the idea of cOSA being uniformly cold. Cascio et al. (43) found cOSA to have virtually no infiltration of CD3+ and CD8+ T cells within the tumors but found both subsets in much higher concentrations in the peri-tumor areas. This aligns well with the definition of “altered” or “excluded” tumors, an intermediate between hot and cold, that have T cells present in tumor margins that are excluded from entering the tumor (38). The presence of distinct immune subtypes with low, intermediate, and high immune infiltrate has already been described in hOSA and is shown to affect response to immunotherapy treatments (44). Each tumor type—cold, altered, or hot—has distinct features that make them more or less likely to respond to a specific treatment, such as checkpoint inhibitors or adoptive cell therapy (38, 44, 45). Based on the available literature, cOSA recapitulates the heterogeneity of immune infiltrates and distinct immune score subtypes seen in hOSA. Still, choosing therapeutics based on levels of immune infiltrates has not yet been explored expressly in cOSA, and further studies are needed to corroborate the use of immune score to predict response to treatment and survival as seen in humans.

## Checkpoint Inhibitors: PD-1/PD-L1

Although beyond the scope of this review and reviewed in detail elsewhere (46, 47), an understanding of the PD-1/PD-L1 pathway is critical to understanding the interactions of T cells with tumor cells as well as other immune cells. PD-L1 is frequently upregulated on tumor cells, and its interaction with PD-1 on immune cells induces tumor tolerance and allows for immune evasion (46). PD-L1 is also found to be expressed on T cells in mouse models with PD-1+ T cells exhibiting multiform interactions that lead to protumor effects (20). Both anticanine PD-1 and PD-L1 therapeutic antibodies have been developed and proven to possess antitumor activity in dogs with cancer (48, 49).

The first study to look at PD-L1 in cOSA did not find expression in samples using IHC, although the study only had three cOSA samples (50). Subsequent studies have found that the majority or all cOSA samples evaluated by IHC express PD-L1 (51, 52). PD-L1 expression in cOSA tumors was likewise consistently found by Cascio et al. (43), whose results show that expression of PD-L1 is associated with resistance to T cell infiltration from the peri-tumor environment to within the tumor, but the study did not evaluate prognostic significance. Although the expression of PD-L1 varies in hOSA, it is consistently associated with TILs. Studies found that PD-L1 is expressed in up to 25% of hOSA tumors and correlates with increased infiltration of PD-1+, CD3+, and CD56+ cells; however, there is no significant correlation to survival (53). A later study found that more than 43% of hOSA harbor PD-L1+ tumor cells with positive correlations to TILs (54). Similar to overall levels of immune infiltration in OSA, the impact of PD-L1 expression in hOSA is conflicting because PD-L1 expression is associated with a negative prognosis secondary to immune dysfunction and also better event-free survival and overall survival because of greater density of TILs and other immune cells (54). Additionally, an increase in PD-L1-expressing tumor-infiltrating immune cells is significantly associated with response to humanized anti-PD-L1 antibody (55), though the specific indications of these biomarkers for response to treatment varies within different cancer types (56). Consequently, further characterization of PD-L1 expressing cells in cOSA is needed for accurate comparison to human studies and investigation of cOSA's sensitivity to PD-1/PD-L1 blockade.

## NK Cells

Even in scenarios in which T cells are present in the TME, cancer cells can suppress MHC-I expression, which is necessary for CD8+ T cells to recognize a target and enact their cytotoxic functions. NK cells, on the other hand, recognize “missing-self” or the lack of MHC-I molecules and can exert their cytotoxic functions in situations in which CD8+ T cells cannot, forming a basis of reasoning for their use in immunotherapies (13). This is seen specifically in hOSA, in which the majority of tumors showed diminished expression of MHC-I, and its downregulation is associated with a worse prognosis (57). NK cells are proven to be capable of lysing hOSA cells (58), and adoptive transfer of NK cells serves as a mechanism to increase the numbers of cytotoxic cells capable of targeting OSA cells *in vivo*. Canine and human NKp46+ NK cells show impressive similarities in expression of natural cytotoxicity receptors and secretion of factors, such as IFN-γ and TNF-α (59). In addition, NKp46+ is not expressed uniformly across NK cells, and its absence correlates with decreased cytotoxicity across species (59). The similarities in both NK cells and OSA in general make dogs an ideal candidate for comparative studies of NK cell infiltrates in OSA.

Mouse models of osteomyelitis with concurrent OSA were early implications of the role of innate immune cells, including NK cells, in the OSA antitumor response (60). Through NK cell depletion, NK cells were found to be critical in OSA tumor growth inhibition (60). One mechanism by which tumors continue to grow in the presence of NK cells may be through overexpression of TGF-β, a potent inhibitor of NK cells. Canine OSA tumors consistently stain positive for TGFβRI and TGFβRII (61), providing a rational for the expansion and transfer of expanded and TGF-β-imprinted NK cells in cOSA therapy (62, 63). Imprinting of NK cells involves prolonged coculture with IL-2 and TGF-β to produce NK cells that are desensitized to the inhibitory effects of TGF-β and thereby capable of prolonged hyperfunctionality with increased cytotoxicity, cytokine production, and longevity. This approach has the potential for novel use in NK immunotherapies (63). In their phase I trial using hypofractionated RT and autologous intratumoral NK cell transfer in dogs with naturally occurring OSA, Canter et al. (64) demonstrate increased progression-free survival in dogs with OSA compared with historical controls. The same group collected tumor specimens from patients in this first-in-dog clinical trial and found that pre- to post-treatment immune-related gene transcript changes varied considerably between dogs (27). NK gene transcripts have significantly less expression of both CD3+ and CD8+ cells in untreated cOSA tumor samples, but there were no patterns of expression that significantly correlated with survival at six months post-treatment in paired samples (27). Intra-tumoral changes in expression of IL-6, a gene linked to cytotoxic lymphocytes, was higher in dogs with prolonged survival though statistical significance may have been limited by the sample size (27). Future clinical trials with increased sample sizes are needed to better evaluate the prognostic value of cOSA tumor-infiltrating NK cells and the therapeutic benefit of NK cell immunotherapy. It should be noted that the full characterization of canine NK cells and their surface markers is still in progress compared with human NK cells and could provide critical information in their use for NK immunotherapies (65). The use of NK cell transfer has not been explored extensively in hOSA, likely due to limiting factors in the sourcing and expansion of NK cells (66, 67), but early successes seen in cOSA can potentially drive translation of NK immunotherapy to clinical trials in humans.

## Conclusion

Osteosarcoma is an aggressive disease for which novel therapeutics are needed, and dogs with spontaneously occurring cancer are a useful model for hOSA studies. Both cOSA and hOSA share extensive similarities, including the frequency and phenotype of immune cells within the TME and peripheral circulation. The OSA TME constitutes a complex web of interactions, especially among NK and T cells, that can be targeted with immunotherapies. OSA tumors in both humans and dogs fall on a spectrum of immune infiltrate levels that correlate with prognosis, express PD-L1 with association to increased TILs, and show sensitivity to NK cell cytotoxicity. The parallels between cOSA and hOSA can be best put to used after filling the gaps in current knowledge regarding the characterization of the cOSA TME and immunotherapies to target it. Future studies in cOSA are needed to characterize NK cells and the expression of PD-1/PD-L1 in TILs as well as to validate the use of immune infiltrates to predict immune response to therapeutics. Increased understanding of intra-tumoral NK and T cells will influence clinical applications of TIL-targeting treatments in both dogs and humans, ultimately leading to better outcomes for patients with OSA.

## Author Contributions

AR formulated the research topic, performed literature review and critical analysis, wrote, and edited the manuscript and figure. SJ, AG, SC, WC, MK, and RR provided comparative oncology expertise and critical review of the manuscript. RC formulated the research topic, provided comparative oncology and immunology expertise, and provided critical analysis and review of the manuscript. All authors contributed to the article and approved the submitted version.

## Funding

This work was supported in part by National Institutes of Health/National Cancer Institute grants U01 CA224166-01 (RC and RR), R03CA252793 (RC), and T32CA251007 (AR and RC).

## Conflict of Interest

The authors declare that the research was conducted in the absence of any commercial or financial relationships that could be construed as a potential conflict of interest.

## Publisher's Note

All claims expressed in this article are solely those of the authors and do not necessarily represent those of their affiliated organizations, or those of the publisher, the editors and the reviewers. Any product that may be evaluated in this article, or claim that may be made by its manufacturer, is not guaranteed or endorsed by the publisher.
